# Mesenchymal Stromal Cells-Derived β2-Microglobulin Promotes Epithelial–Mesenchymal Transition of Esophageal Squamous Cell Carcinoma Cells

**DOI:** 10.1038/s41598-018-23651-5

**Published:** 2018-04-03

**Authors:** Junjie Wang, Weilin Yang, Tao Wang, Xiaoyong Chen, Jiancheng Wang, Xiaoran Zhang, Chuang Cai, Beilong Zhong, Jiabin Wu, Zhenguang Chen, Andy Peng Xiang, Weijun Huang

**Affiliations:** 1 Center for Stem Cell Biology and Tissue Engineering, The Key Laboratory for Stem Cells and Tissue Engineering, Ministry of Education, Sun Yat-Sen University, Guangzhou, China; 2 Department of Cardiothoracic Surgery of East Division, The First Affiliated Hospital, Sun Yat-sen University, Guangzhou, Guangdong China; 3 Department of Thoracic Surgery, The First Affiliated Hospital, Sun Yat-sen University, Guangzhou, Guangdong China; 4 Lung Cancer Research Center of Sun Yat-sen University, Guangzhou, Guangdong China; 5 Department of Thoracic Surgery, The Fifth Affiliated Hospital, Sun Yat-sen University, Zhuhai, Guangdong China; 6 Department of Biochemistry, Zhongshan School of Medicine, Sun Yat-Sen University, Guangzhou, Guangdong China; 7 The Biotherapy Center, The Third Affiliated Hospital, Zhongshan School of Medicine, Sun Yat-Sen University, Guangzhou, Guangdong China

## Abstract

Mesenchymal stromal cells (MSCs) have been considered as one of the pivotal type of cells composing the tumor microenvironment. Although contact-dependent mechanisms and paracrine factors are thought to collaborate in governing the MSCs-based effects on tumors progression, the underlying mechanisms remain largely unknown. In particular, the involvement of MSCs-derived cytokines in the epithelial–mesenchymal transition (EMT) of esophageal squamous cell carcinoma (ESCC) has not been clarified. In this study, we observed that β2-Microglobulin (B2M) is highly expressed in MSCs but scarcely in ESCC cells. Based on the previously described EMT promoting effect of B2M, we investigated the *in vitro* effect of MSCs-derived B2M on the EMT of ESCC cells, and discovered its subsequent enhancing effects on cell mobility and tumor-initiation. Further xenograft transplantation experiments confirmed the *in vivo* induction of tumor-initiation by MSCs-derived B2M. Noteworthy, we showed that the B2M expression positively correlated with poor prognosis. The fact that B2M is primarily expressed by the stroma of the ESCC tissue strengthens our hypothesis that in ESCC, MSCs-derived B2M promotes tumor-initiation and invasion via enhancing EMT, resulting in an adverse prognosis for the patients. Our results will be valuable for the prediction of the development and treatment of ESCC.

## Introduction

Esophageal squamous cell carcinoma (ESCC) is one of the most aggressive and lethal malignant disease with a 5-year survival after esophagostomy^1,2^. Although advances in diagnosis and treatment of ESCC have been made in recent years, the overall survival rate of patients with distant metastases has not changed significantly in the last decade^3–5^. Hence it should be encouraged to study the mechanism of metastasis and recurrence of ESCC to develop new therapeutic strategies.

As important components of the tumor microenvironment, increasing evidence indicates that tumor-associated fibroblasts (TAFs) are significant regulators of tumor progression and metastasis^6,7^. The origin of TAFs is poorly understood. Mesenchymal stromal cells (MSCs) have been reported to be recruited into the tumors, where they proliferate and acquire the TAF-like phenotype^8^. There is growing evidence to corroborate that cells immuno-phenotypically characterized as MSCs can be defined as TAFs^9,10^. Therefore, MSCs would be a useful tool to investigate the interaction between tumors and TAFs. It has been recognized that MSCs/TAFs affect tumor development through their paracrine effects, but their secreted mediators and underlying mechanisms are still largely unexplored.

β2-Microglobulin (B2M), a 11 KDa non-glycosylated protein, is encoded by a well-known housekeeping gene^11–13^. B2M is expressed by all nucleated cells to form a small invariable light chain subunit of the major histocompatibility complex (MHC) class I antigen on the cell surface^14^. In addition, soluble B2M could be detected in extracellular fluid^11,15^. The levels of soluble B2M have been reported to increase in a number of liquid and solid tumors^16^, and could be regarded as a prognostic factor for some malignancies^17,18^. Mechanistically, B2M is able to mediate tumorigenesis, angiogenesis, metastasis and osteomimicry^19–21^. Since B2M has been reported to be highly-expressed in MSCs and decreased in ESCC tissues^22,23^, we speculated that MSCs/TAFs might regulate ESCC development via B2M.

In this study, we revealed that MSCs-derived B2M significantly induced epithelial-to-mesenchymal transition (EMT) in ESCC cells, and observed its subsequent enhancing effects on cell mobility and tumor-initiation. Further xenograft transplantation experiments confirmed the *in vivo* enhancing tumor-initiation effect induced by MSCs-derived B2M. Finally, we found that the expression of B2M correlated with poor prognosis. Collectively, our results strengthen our hypothesis that in ESCC, MSCs-derived B2M promotes tumor-initiation and invasion via enhancing EMT, resulting in a poor clinical outcomes for the patients.

## Results

### B2M is highly-expressed in MSCs and low in ESCC cells

**Figure 1:**
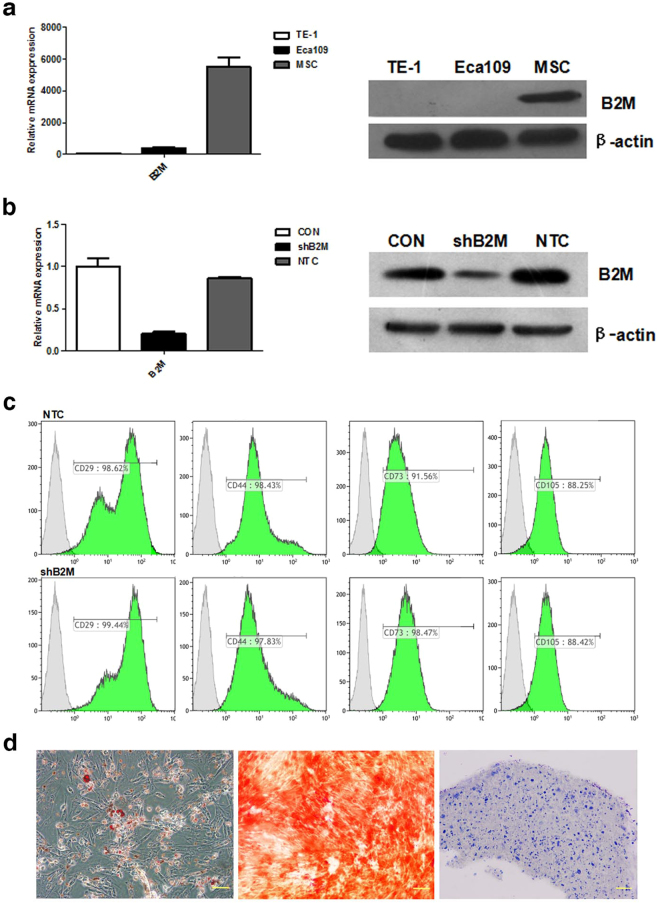
High expression of B2M in MSCs and MSC^shB2M^ retained the multipotent differentiation ability of MSCs. (**a**) MSCs have a high expression of B2M while esophageal cancer cells (Eca109 and TE-1) barely express B2M, both at the mRNA (qRT-PCR; left panel) and at the protein (Western blot; right panel) level. (**b**) Construction of control RNAi (MSC^NTC^) and B2M RNAi knockdown (MSC^shB2M^) MSC cell lines, showing over 79% B2M knocking down effect by B2M RNAi, at both the mRNA (qRT-PCR; left panel) and the protein (Western blot; right panel) level. (**c**) The results of the flow cytometry analysis revealed that the MSC^NTC^ and the MSC^shB2M^ cells shared specific surface markers with normal MSCs (CD29, CD44, CD73 and CD105). (**d**) Light microscopy images of MSC^shB2M^ cell samples assayed for adipogenic (Oil Red O staining), osteogenic (Alizarin Red S staining), and chondrogenic (toluidine-blue staining) differentiation (left, middle, and right panels, respectively). The imaging results revealed that the MSC^shB2M^ retained its multipotency. CON: original bone marrow MSCs; Scale bars: 100 μm.

Previous studies have shown that the expression of B2M was high in MSCs and reduced in ESCC tissues^22,23^. Consistent with these reports, we observed high B2M expression in the human bone marrow MSCs, both at the RNA and the protein level, and low B2M expression in the ESCC cell lines (Eca109 and TE-1; Fig. 1a and Supplementary Fig. S2).

To investigate whether MSCs-derived B2M could be contributing to the ESCC development, we generated MSCs with B2M knockdown by RNA interference, which were designated as MSC^shB2M^. The knockdown effect of B2M was assessed by quantitative polymerase chain reaction (qPCR) which confirmed that there was a 79% reduction of B2M expression compared to the MSCs transfected with the empty vector control sequences (MSC^NTC^) (Fig. 1b). Western blotting analysis demonstrated that the expression of B2M was significantly declined in the whole-cell lysates of MSC^shB2M^ (Fig. 1b and Supplementary Fig. S5). Both MSC^NTC^ and MSC^shB2M^ expressed the same type of surface markers (including CD29, CD44, CD73 and CD105; Fig. 1c and Supplementary Fig. S3), and had similar differentiation capacity (including osteogenic, adipogenic and chondrogenic differentiation; Fig. 1d). These results indicated that B2M knockdown could not affect the fundamental properties of MSCs.

### MSCs-derived B2M promotes epithelial-mesenchymal transition and enhances mobility of ESCC cells *in vitro*

**Figure 2:**
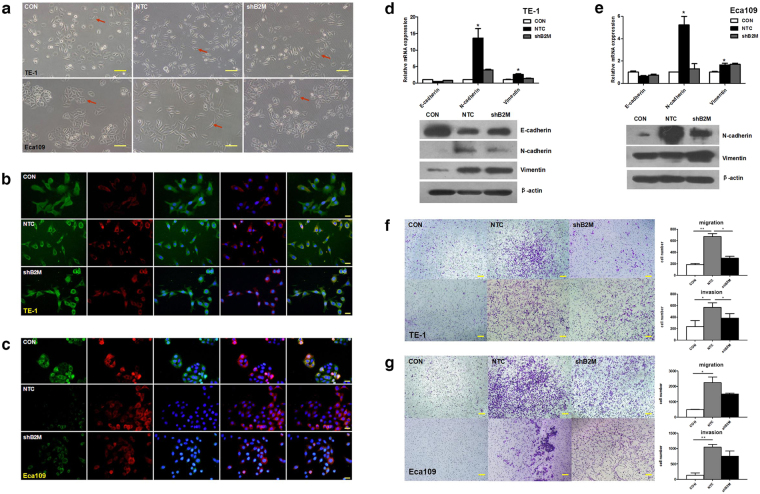
MSCs-derived B2M promotes epithelial-mesenchymal transition and enhances mobility of ESCC cells *in vitro*. (**a**) Morphological appearance of esophageal cancer cells treated with conditioned medium of MSC^NTC^ and MSC^shB2M^ suggested MSCs-derived B2M promotes EMT *in* vitro. In mesenchymal status, TE-1 present morphological changes from cobblestone-like to cord-like cells with sharp protuberance while Eca109 present incompact structure instead of clustered ‘islets’. Red arrows indicate representative morphological changes of tumor cells. (**b,c**) Immunofluorescence imaging revealed increased expression of N-cadherin (red) accompanied with decreased expression of E-cadherin (green) after treatments with MSC^NTC^-CM but not with MSC^shB2M^-CM in (**b**) TE-1 and (**c**) Eca109 cells. Nucleuses were immunostained with DAPI (blue). From left to right the images show the staining results for E-cadherin, N-cadherin, E-cadherin + DAPI, N-cadherin + DAPI, and Merge. (**d,e**) The qRT-PCR (upper panel) and western blot (lower panel) results showed elevated N-cadherin and vimentin with down-regulated E-cadherin in TE-1(**d**) and Eca109 (**e**) cancer cells after treated with MSC^NTC^-CM in contrast to the MSC^shB2M^-CM. (**f**) The number of the TE-1 cells in the migration (upper panel) and the invasion (lower panel) assays after treatment with MSC^NTC^-CM was significantly increased compared with the negative control, while RNAi against B2M (MSC^shB2M^) partially blocked this effect. (**g**) Similar migration and invasion results were observed in the Eca109 cells. Data is expressed as mean ± S.D. of three independent experiments. Error bars indicate S.D. (n = 3); *p < 0.05, **p < 0.01; CON: tumor cells untreated with conditioned medium; Scales bars: 100 μm.

Epithelial-mesenchymal transition (EMT) is a tumor progression associated process^24^. Considering the importance of EMT in tumor development, we investigated whether MSCs-derived B2M could influence the EMT process of ESCC cells *in vitro*. MSCs-conditioned media (CM) was harvested, filtered, and concentrated 25–30 times to be used in our study. Indeed, we observed significant morphological changes in ESCC cells treated with MSC^NTC^-CM. In particular, TE-1 cells acquired a more elongated cell shape instead of the cobblestone appearance, while Eca109 cells lost conspicuous cell-cell contact (Fig. 2a). Besides, immunofluorescence assays showed an apparent elevation of N-cadherin and down regulation of E-cadherin (Fig. 2b and c), which indicates that the cells have undergone or undergoing through the EMT process. The results of qPCR and western blot analysis confirmed the changes of these EMT-related markers (Fig. 2d,e and Supplementary Fig. S6). We did not achieve E-cadherin detection in the Eca109 cells by western blot possibly due to the relatively low expression of this protein. Furthermore, it might be hard to find out the changes of vimentin in Eca109 because its expression is appreciable, which could be contribute to the poorly differentiated status of tumor cells^25^. In contrast, induction of EMT-related markers by MSC^shB2M^-CM was predominant restrained in the ESCC cells (Fig. 2a–e). These results suggested that B2M expression in MSCs played an important role in the EMT induction of ESCC cells.

EMT is considered to be associated with tumor cell mobility, which may play an important role in metastasis^26^. Hence, transwell systems were used to investigate the mobility of ESCC cells upon MSCs-CM treatment. The results showed that ESCC cells treated with MSC^NTC^-CM exhibited a 3- to 10-fold increased migration/invasion capability when compared with the negative control. Notably, MSC^shB2M^-CM restored the migration and invasion of ESCC cells, especially in TE-1 cells (Fig. 2f and g). The results indicated that the MSCs-derived B2M contributes to the increased mobility of ESCC cells which might be associated with the process of EMT.

Previous studies have shown that B2M-driving EMT occurs in conjunction with the consistent activation of STAT3, Snail, LIV-1, and RANKL^12^. Hence, we inspected several components of the JAK2/STAT3 pathway to explore whether the molecular mechanism underlying the EMT was triggered by MSCs-derived B2M. Western blot analysis showed that the expression of the EMT-related markers, triggered by MSCs-CM, could be inhibited by AG490 (JAK-2 protein tyrosine kinase inhibitor), indicating that the JAK2/STAT3 pathway involved in the EMT process. However, down-regulating B2M of MSCs barely affected the phosphorylation of STAT3 (Supplementary Fig. S7). It suggested that B2M may not directly regulate STAT3.

### MSCs-derived B2M contributes to ESCC cells acquisition of stemness *in vitro*

**Figure 3:**
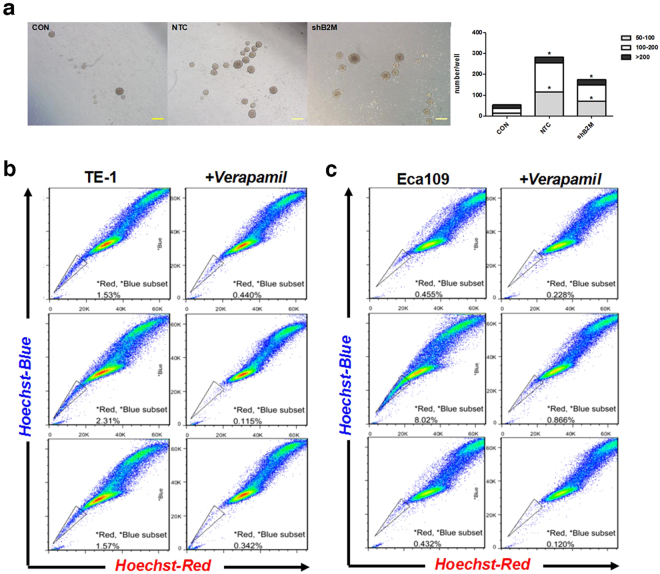
MSCs-derived B2M contributes to ESCC cells acquisition of stemness *in vitro*. (**a**) MSC^NTC^-CM increased the spheroids formation ability of TE-1 cells, while down-regulation of B2M in MSCs significantly suppressed this effect. Spheroids were photographed, measured and sorted into groups in accordance with their diameter. The calculated numbers of spheroids in each group are depicted in the bar graph (right panel).CON: TE-1 cells untreated with conditioned medium. (**b,c**) Freshly isolated TE-1 and Eca109 cells were enriched for Hoechst^low^ SP cells after treatment with DMEM control medium (upper panel), MSC^NTC^-CM (middle panel) and MSC^shB2M^-CM (lower panel). The proportion of SP cells was increased significantly under MSC^NTC^-CM treatment, compared with the negative control, and down-regulation of B2M restored the SP cells proportion in ESCC cells. Data were expressed as mean of three independent experiments. *p < 0.05, Scale bars: 200 μm.

Recent studies have indicated that the process of EMT could generate cells with properties of stem cells^27^. We further investigated whether MSCs-derived B2M could affect the acquired stem-like properties of ESCC cells during the EMT process. It is well recognized that tumor stem-like cells could undergo anchorage-independent growth, allowing the formation of multi-cellular tumor spheroid colonies^27^. Therefore, we performed spheroids formation assay and counted the 3D tumor spheres in this study. The spheres were grouped into three populations based on their diameter (50 to 100 μm, 100 to 200 μm and more than 200 μm). Results showed that both MSC^NTC^-CM and MSC^shB2M^-CM could significantly promote the formation of tumor spheres including their quantity and dimensions, when compared to the control group (without CM treatment). The anchorage-independent growth ability of ESCC cells under MSC^NTC^-CM was significantly stronger than that under MSC^shB2M^-CM treatment (*p* = 0.004; cells per well with diameter 50 to 100 μm: 116.7 ± 5.8 versus 74.3 ± 7.3, 100 to 200 μm: 138.1 ± 9.8 versus 79.0 ± 2.9, and more than 200 μm: 30.0 ± 2.4 versus 22.3 ± 5.4; Fig. 3a and Supplementary Fig. S4).

To verify this discovery, analysis of side population (SP) cells was employed in our study. SP cells are typically identified according to their ability to efflux Hoechst 33342 dye through high-expressed adenosine triphosphate-binding cassette membrane transporters, which could be inhibited by verapamil^28^. Since these transporters are also highly-expressed in stem cells, SP cells are thought to be highly enriched in tumor stem-like cells^29^. The results showed that the proportion of SP cells was increased considerably in ESCC cells under MSC^NTC^-CM treatment compared with the negative control (TE-1: 2.195% versus 1.09%, Eca109: 7.154% versus 0.227%; Fig. 3b and c). The SP cells proportion in ESCC cells under MSC^shB2M^-CM treatment was comparable to the negative control (TE-1: 1.228%, Eca109: 0.312%). All these results confirmed that B2M of MSCs is essential in promoting tumor cells acquiring stem cell-like properties.

### MSCs-derived B2M has limited influence on cell proliferation and induces drug resistance in ESCC cells *in vitro*

**Figure 4:**
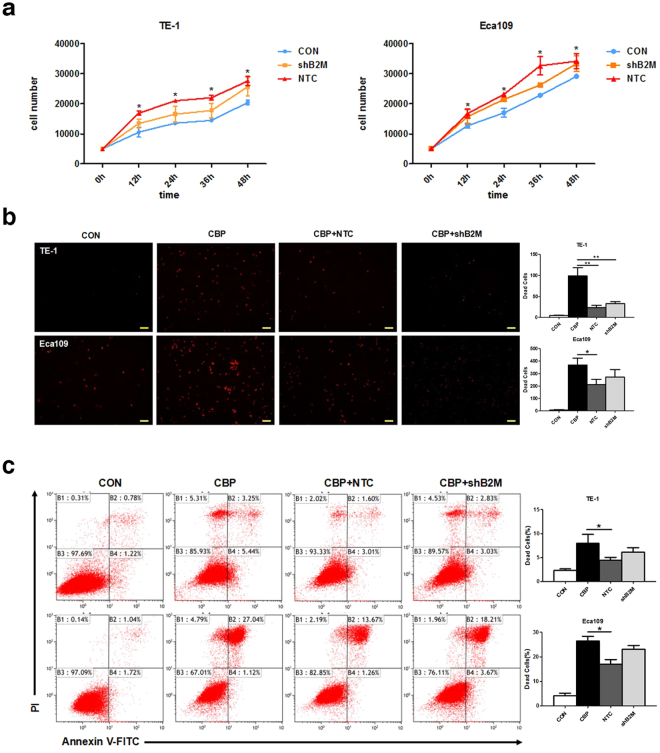
MSCs-derived B2M has limited influence on cell proliferation and induces drug resistance in ESCC cells *in vitro*. (**a**) MSC^NTC^-CM significantly promoted the proliferation of ESCC cells compared with the negative control. Down-regulation of B2M partially blocked this increase in proliferation. CON: tumor cells untreated with conditioned medium. (**b**) ESCC cells were exposed to carboplatin (20–40 μg/ml, 24 h) with or without conditioned medium then examined for dead cell numbers; red luminescence indicated the dead cells. MSC^NTC^-CM could induce drug resistance to apoptosis while MSC^shB2M^-CM showed a moderate effect. CON: tumor cells untreated with carboplatin and conditioned medium. (**c**) Apoptotic cells were detected by flow cytometry assay with double staining by Annexin V-FITC/Propidium Iodide in TE-1 (upper panel) and Eca109 (lower panel) cells. CON: tumor cells untreated with carboplatin and conditioned medium. Quantification of the apoptotic rate and statistical comparisons are presented in the bar charts in the right panels. Data were expressed as mean ± S.D. of three independent experiments. Error bars indicate S.D. (n = 3); *p < 0.05, **p < 0.01; Scale bars: 100 μm.

Tumor cells undergoing EMT might enhance their viability^30^. We further tested if MSCs-derived B2M influences the viability of ESCC cells. Cell proliferation assay with cell counting kit-8 was performed in our research. The results of counting the cell numbers every 12 hours suggested that MSC^NTC^-CM could significantly promote the proliferation of ESCC cells, exhibiting a 1.5- to 3-fold increase compared with the untreated group (TE-1: *p = *0.004 & Eca109: *p = *0.011, Fig. 4a). Although MSC^shB2M^-CM can promote cell proliferation, the induction effect was partially descended comparing to the MSC^NTC^-CM treated group (Fig. 4a), which could either indicate that additional factors are possibly involved and/or the residual B2M (incomplete RNAi effect) in the CM could have induced the small increase in cell proliferation.

Cisplatin is a first-line chemotherapeutic drug for the treatment of ESCC^31^. Apoptosis may play an important role in cisplatin sensitivity of tumor cells via caspase-dependent and -independent mechanisms^32^. In clinical trials, chemo-resistance to cisplatin limits its treatment success which often leads to unfavorable prognosis^33^. Hence we investigated whether MSCs-derived B2M contributes to the resistance to apoptosis triggered by chemotherapeutic drugs. The number of dead tumor cells counted by LIVE/DEAD Viability/Cytotoxicity Kit indicated that MSC^NTC^-CM could significantly enhance the drug-resistant ability of ESCC tumor cells (Eca109: 211.8 ± 42.1 versus 369.4 ± 54.9, *p = *0.03; TE-1: 23.2 ± 5.6 versus 98.9 ± 19.6, *p = *0.001; Fig. 4b). The MSC^shB2M^-CM treatment presented a similar but attenuated effect (Eca109: 272.4 ± 60.9 versus 369.4 ± 54.9, *p = *0.053; TE-1: 32.8 ± 4.9 versus 98.9 ± 19.6, *p = *0.007; Fig. 4b). Flow Cytometry analysis of Annexin V and propidiumiodide staining further confirmed these results (Eca109: 17.06 ± 1.84 versus 26.48 ± 1.85 for MSC^NTC^-CM, *p = *0.043, 23.04 ± 1.59 versus 26.48 ± 1.85 for MSC^shB2M^-CM, *p = *0.14; TE-1: 4.47 ± 0.59 versus 7.99 ± 1.83 for MSC^NTC^-CM, *p = *0.039, 6.12 ± 0.93 versus 7.99 ± 1.83 for MSC^shB2M^-CM, *p = *0.11; Fig. 4c). It suggested that MSCs-CM could affect the ESCC cells’ chemo-resistance to carboplatin, but B2M had a minor contribution to this process.

### MSCs-derived B2M enhances tumor development *in vivo*

**Figure 5:**
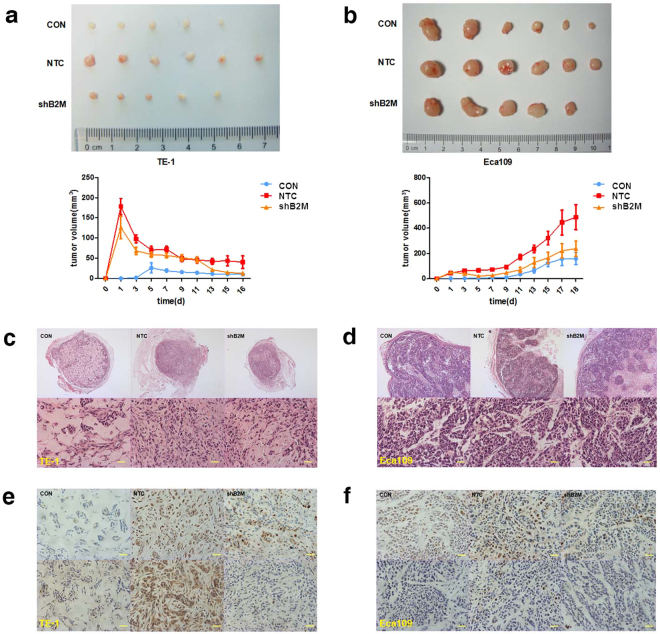
MSCs-derived B2M enhances tumor development *in vivo*. (**a,b**) Representative macroscopical images of fresh BALB/c nude mice tumor tissues (upper panels) and the dynamic tumor growth curves in the TE-1 (**a**) and the Eca109 (**b**) cell group (lower panels). MSC^NTC^ contributed to the enhancement of the xenograft tumor formation rate of the ESCC cells compared with the negative control, and knockdown of B2M reversed this tendency. The average tumor volume in the MSC^NTC^ group was significantly larger than in mice injected with ESCC cells only, while the tumor volume in the MSC^shB2M^ group was much smaller than that in the MSC^NTC^ group until the sacrifice of animals. (**c, d**) H&E staining results showed the pathological structure of xenograft tumor section formed by TE-1 (**c**) and Eca109 (**d**) cells, including representative image of intact section (upper panels) and regional details (lower panels). (**e,f**) Immunohistochemical staining of TE-1 (**e**) and Eca109 (**f**) cells from the xenograft tumor tissue was performed using anti-PCNA (upper panel) and anti-Ki67 (lower panel) polyclonal antibodies. Tissue sections of MSC^NTC^ group displayed more positive signals compared with the negative control and the MSC^shB2M^ group. Data were expressed as mean ± SEM of three independent experiments. Error bars indicate SEM (n = 6); *p < 0.05; CON: mice transplanted with tumor cells alone; Scale bars: 100 μm.

Our results of *in vitro* experiments indicated that B2M of MSCs could promote tumor cells acquiring stem cell-like properties, which may enhance their initiating capability. Hence, to clarify the potential effect on tumor development of MSCs-derived B2M *in vivo*, we subcutaneously injected 1 × 10^6^ ESCC cells either alone or in combination with the same amount of MSCs into nude mice and observed the formation of xeno-transplanted tumors. As showed in Fig. 5a and b, MSC^NTC^ could significantly enhance the tumor formation ability in nude mice compared with the negative control, based on the tumor volume results (Eca109: 486.6 ± 240.8 mm^3^ versus 158.0 ± 115.9 mm^3^ on day 18 after xenograft, *p = *0.013; TE-1: 40.5 ± 38.2 mm^3^ versus 10.0 ± 5.7 mm^3^ on day 16 after xenograft, *p = *0.041). Meanwhile, the induction effect of MSC^shB2M^ was substantially weakened (Eca109: 238.0 ± 148.6 mm^3^ versus 158.0 ± 115.9 mm^3^ on day 18 after xenograft, *p = *0.32;TE-1: 12.8 ± 11.1 mm^3^ versus 10.0 ± 5.7 mm^3^ on day 16 after xenograft, *p = *0.059). In order to exclude the volume interference of the co-injected MSCs, we performed immunohistochemistry analyses of xenograft tumor sections retrieved from animals. The components of all xenograft tumors were mainly comprised of cancer cells (Fig. 5c and d). Specifically, MSC^shB2M^ *in vivo* seemed to promote TE-1 cells formation into a well-organized tumor structure with decreased cavities (Fig. 5c). Besides, we also observed an increased expression of the cell proliferation markers, PCNA and Ki67, in the MSC^NTC^ group and an attenuated expression in the MSC^shB2M^ group (Fig. 5e and f). These results demonstrate that MSCs-derived B2M might contribute to an enhanced tumor-initiating capability of ESCC *in vivo*.

### B2M expression correlates with poor prognosis

**Figure 6:**
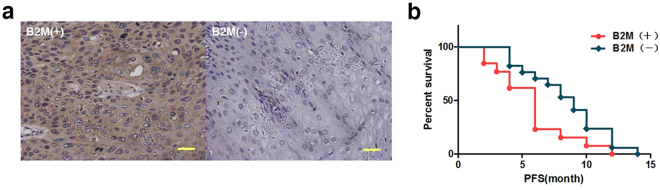
High expression of B2M in patients suggests poor survival. (**a**) Representative images indicate positive (left panel) and negative (right panel) expression of B2M in clinical samples. (**b**) Kaplan-Meier analysis of B2M expression in ESCCs: Patients with high expression of B2M have shorter PFS (*p* < 0.05) after operation. Scale bars: 100 μm.

We further performed a comprehensive analysis of samples from 30 ESCC patients after resection, to determine if B2M expression in tumors correlated with prognosis. Progression-free survival (PFS) is often used to evaluate the recurrence rate of cancer, which has a promised linkage to EMT induced metastasis compared with overall survival (OS)^34^. After PFS analysis and summarizing the B2M staining results and the related clinical information (Fig. 6a and Supplementary Table S3), we found that B2M expression in tumors was associated with the poor prognoses of ESCC patients (*p = *0.039; Fig. 6b). High expression of B2M predicted a shorter PFS for patients, which suggested that B2M could be possibly considered as a potential metastasis indicator in clinical prognosis. However, further investigation to reveal the important role of MSCs-derived B2M in tumorigenesis is mandatory.

## Discussion

MSCs are important components of the tumor microenvironment, which have been considered as origins of TAFs^35^. Previous studies indicated that MSCs migrate to primary tumor sites by chemotaxis^36^, and execute immunosuppression, regulate the EMT process, and contribute to establishment of cancer stem cell niches in various types of cancer^37–39^. Contact-dependent mechanisms and paracrine factors are thought to collaborate in governing the MSC/TAF-based effects on tumors^40,41^. EMT is a tumor progression associated process as tumor cells undergoing EMT might lose epithelial markers and acquire mesenchymal phenotypes^24^. This transition enhances their invasive capacity, assigns carcinoma stem cell properties to the tumor cells^42–44^, as well as elevates their viability and resistance to apoptosis^30^. Our results showed that MSCs-CM could induce a significant EMT phenotype, thereby increase migration, tumor-initiating capability, cell viability and apoptosis resistance of ESCC cells. Recent studies showed that MSCs could affect tumor progression through secreted factors influencing immunoregulation (IL6, IL10, TGF-beta and PGE2), tumor cell survival (STAT3, MAPK, PARP1 and caspase3), tumor angiogenesis (VEGF, bFGF, IL6, IL8, angiopoietin), and tumor cell motility/metastasis (CCL5, CXCL12 and IGF1)^45–47^. Our findings were consistent with these studies and added to the growing evidences for the crosstalk between MSCs/TAFs and tumor progression through paracrine effects. However, blocking individually any of the paracrine molecules did not completely abrogate the role of MSCs/TAFs in tumor progression, indicating that these factors act synergistically or that additional, yet unidentified, mediators might be involved in the EMT process.

B2M on the cell surface can interchange with its free soluble form in the body fluids because there is no direct attachment on the cell membrane^14,15^. Previous studies and our results showed that B2M expression was relatively low in ESCC cells^23^. Josson *et al*. reported that B2M overexpression could drive EMT and promote the growth, invasion, and metastasis of human prostate, breast, lung, and renal cancer cells *in vitro* and *in vivo*^16^. Also, elevated B2M levels in the urine and the serum have been demonstrated to negatively correlate with the survival of patients with prostate cancer^48^. Our study clearly shows that B2M is important for MSCs to promote the EMT process and enhance the mobility of ESCC cells *in vitro*. It suggested that the expression level of B2M in the stroma of the ESCC tissue might be an important parameter of prognosis evaluation.

Recent studies revealed that induction of EMT could convert cells into cells with stem-like properties^27,49^. This information provides a crucial link between the metastatic traits and the initiating capability of tumor cells to undergo EMT. Cancer cells with stem-like properties are recognized as cancer stem cells (CSCs), which can self-renew and divide by asymmetric cell division to generate cellular heterogeneity in the originating tumor. CSCs also have been shown to exhibit an increased level of “quiescence” and resistance to chemo-drugs or nuclei dyes^50^. We found *in vitro* and *in vivo* that MSCs-derived B2M can enhance the tumor-initiating capability of ESCC cells. Considering that tumor-initiating capability is related to distant metastases, it is logical to further suggest that B2M might be a biomarker and/or drug target of distant metastases of ESCC.

As many epithelial tumors, ESCC contains heterogeneous cell populations. Hence we employed two human ESCC cell lines, Eca109 (poorly differentiated)^51^ and TE-1 (well differentiated)^52^, as the model system to investigate if the effects of the MSCs-derived B2M on ESCC cells are general. The heterogeneity of tumor cells representing different stages of tumorigenesis could affect the outputs of crosstalk between MSCs/TAFs and tumor, even lead to controversial results^53,54^. For instance, MSCs could inhibit the invasion of U87 cells but enhance the invasion of U373, both of which are glioblastoma cell lines^55^. Although the results of the two cell lines in our study were quantitatively slightly different (possibly due to the incomplete knockdown of B2M by RNAi), the similar effects suggested that B2M is essential for MSCs to trigger EMT by enhancing mobility and acquisition of stem-like properties in ESCC cells *in vitro* and *in vivo*. So this both added to the evidence and helped to confirm that B2M should be valuable for the prognosis and treatment of ESCC.

The molecular mechanism of the secreted B2M on promoting EMT is poorly understood, since its receptor is still ambiguous. Nomura *et al*. first showed that B2M could activate cAMP-dependent PKA activity by binding to the seven-transmembrane G protein-coupled receptor (GPCR)^20^. Josson *et al*. reported that hemochromatosis (HFE) protein, a non-classical MHC class I member, interacts with B2M to regulate iron homeostasis and control EMT of cancer cells via interaction with TFRC 1 (transferrin receptor complex 1) and suggested that B2M/HFE interactions are important for the secreted B2M-mediated EMT^16^. Zhau and Nomura *et al*. reported that B2M-transfected cancer clones expressing indicative EMT markers consistently show increased levels of activated STAT3, Snail, LIV-1, and RANKL^12,56^. Actually, it is widely accepted that MSCs could mediate EMT/metastasis of tumor through JAK/STAT signaling. Recent studies reported that JAK/STAT signaling was involved in the promotion effect of MSCs on EMT/metastasis in colorectal tumor^57^ and lung cancer^41^. Torsvik and Bjerkvig reviewed MSCs signaling involved in cancer progression and concluded that MSCs-secreted IL6 could promote initiation and EMT/metastasis of tumor via activation of JAK/STAT pathway^58^. Hence, we speculate that JAK/STAT might be involved in EMT medicated by MSCs-derived B2M. Although our results suggested the possible involvement of JAK/STAT3 pathway in the EMT process, there were no definitive results to confirm any direct interaction involving B2M. Since we observed a decreased expression of snail in MSC^shB2M^ group (Supplementary Fig. S7), we inferred that B2M may facilitate the nucleus location of *p*-STAT3 and/or affect downstream DNA transcription as a transcription factor which mechanism need to be further elucidated.

The prognostic impact of B2M has been well demonstrated in different types of cancers, but its relationship with ESCC has not been thoroughly explored. Tanaka *et al*. reported the association between B2M expression and endpoint evaluation of esophageal cancer patients, but their definition of the expression levels of B2M seemed to be crude and arbitrary^18^. Noteworthy, B2M expression in the tumor samples of our study showed a significant positive correlation with tumor progression. This result was obtained through the Kaplan–Meier analysis of PFS (progression-free survival) in ESCC patients, who received first-line chemotherapy after resection. Comparing to traditional end point OS (overall survival), PFS could be considered a valid surrogate in advanced cancer therapy assessment which requires shorter follow-ups and also possesses the intrinsic advantage of assessing the time of tumor development^59,60^. Considering that B2M is primarily expressed by the stroma of the ESCC tissue, our results further strengthen our hypothesis that in ESCC, MSCs-derived B2M promotes tumor-initiation and invasion via enhancing EMT, resulting in an adverse prognosis for the patients. In order to highlight its value for the prognosis and treatment of ESCC, further research basing on multicenter, large sample, the long-term clinical observation should be needed to confirm this conclusion.

In conclusion, our study implied the potential role of MSCs-derived B2M in the ESCC development and further confirmed the reciprocity between MSCs and tumor. Further studies should be focused on clarifying the details and mechanisms of biological function of soluble B2M to illuminate the clinical significance of MSCs-derived B2M in ESCC. Besides, MSC-based therapy have attracted great interest as a new treatment in lots of refractory or incurable diseases. Our study also suggested that the malignant effects of MSCs should be fully considered.

## Methods

### Ethical statement

Written informed consent was obtained from all the participants, and the study protocol was approved by the ethics committee of Sun Yat-sen University (SYSU), Guangzhou, China. All animal experiments were approved by the Animal Care and Use Committee and conducted in accordance with the official recommendations of the Care and Use Laboratory Animals of Sun Yat-sen University, Guangzhou, China.

### Cell culture and preparation of conditioned medium

Human bone marrow-derived MSCs were obtained from healthy donors, according to the ethical guidelines of the Sun Yat-sen University, Guangzhou, China. The human esophageal cancer cell line TE-1 and Eca109 were purchased from the Chinese Academy of Sciences (Shanghai, China). Knockdown B2M MSCs were generated in our lab as follows. The tumor cells were maintained in Dulbecco’s modified Eagle medium (DMEM) supplemented with 10% FBS, 100 U/ml penicillin, and 100 μg/ml streptomycin, at 37 °C in a humidified atmosphere containing 5% CO_2_. MSCs were cultured in the same medium supplemented with 2 ng/L bFGF (Gibico,Waltham,MA,USA). To prepare conditioned media (CM), MSCs were grown to 90% confluence in 75 cm^2^ flasks in DMEM/10% FBS. Then the medium was discarded, washed with 0.1% PBS twice, and the MSCs were further cultured in serum and antibiotics free DMEM for 48 h. The medium was then collected and filtered through 0.45 μm filters and finally concentrated (1:30) by the Amicon Ultra centrifugal filter units (Millipore, Billerica, MA, USA). Aliquots of concentrated conditioned medium were stored in −80 °C for further research.

### Plasmid construction and cell transfection

Briefly, B2M shRNA was sub-cloned from the pLL3.7-cmv plasmid and inserted into a lentiviral vector system. Then the constructed vector and the packing plasmid were co-transfected into 293 T cells using Lipofectamine 2000 (Invitrogen, Grand Island, NY, USA). 24 hours after transfection, the supernatant containing the viruses was concentrated and used to infect MSCs. MSCs were cultured in 75 cm^2^ flasks at a density of 6 × 10^5^ for 24 h to reach a sub-confluent status. The MSCs were then infected with B2M-specific shRNA or a vehicle shRNA control with polybrene (Santa Cruz Biotechnology, Dallas, TX, USA), according to the manufacturer’s instructions. The specific sequences of B2M shRNA are provided in Supplementary Table S1. After transfection, MSCs were passaged and B2M gene expression was tested at both the mRNA and the protein level; only MSCs with over 79% B2M knockdown affect were used in subsequent experiments (Supplementary Fig. S1).

### RNA isolation and quantitative Real-time PCR (qRT-PCR)

Total RNA was isolated with the TRIzol Reagent, and cDNA was synthesized using the RNA RT-PCR Kit (Thermo scientific, Waltham, MA, USA). After that, real-time PCR was performed using the Roche LightCycle 96 Real-Time PCR system and the SYBR Premix Ex Taq II Kit (Roche, Basell, Switerland). All the procedures were performed according to the manufacturer’s protocols. The specific quantitative Real-time PCR primer sequences are provided in the Supplementary Table S2. All the reactions were performed in triplicate using 2 μl samples containing 20–50 ng of complementary DNA.

### Antibodies and Western blot analysis

Whole cell extracts were prepared by lysing the cells in RIPA buffer (1 × PBS, 0.5% sodium deoxycholate, 1% NP-40, 0.1% sodium dodecyl sulfate supplemented with freshly added 10 mM β-glycerophosphare, 10 mM NaF, 1 mM phenylmethylsulfonyl fluoride, 1 mM sodium orthovanadate, pH 7.4) containing a cocktail of protease and phosphatase inhibitors. Equal amounts of protein (20–40 μg) were separated by SDS–PAGE and transferred to PVDF membranes. The membranes were probed with β2-microglubin (ab54810, abcam,Cambridge, MA, USA), Vimentin (ab45939, abcam), E-cadherin (sc7870, SantaCruz Biotechnology). N-cadherin (610920, BD Transduction Laboratories, Franklin, MI, USA), snail (cst3879,Cell Signaling Technology), *p*-STAT3 (#9131, Cell Signaling Technology, Danvers, MA) or β-actin primary antibodies (AC15, Sigma-Aldrich, St Louis, MO, USA). Anti-rabbit and Anti-mouse secondary antibodies were purchased from Proteintech (SA00001–2, ProteinTech, Rosemont, IL, USA) and (SA00003–1). The target proteins were detected using the Millipore Immobilon ECL Liminol Reagent (WBLUC0100, Millipore) and film exposure. The antibodies used in Flow Cytometry were CD29 (555005, BD Pharmingen), CD44 (553134, BD Pharmingen), CD73 (561260, BD Pharmingen), and CD105 (562408, BD Pharmingen).

### Adipogenic, Osteogenic and chondrogenic differentiation assays

For the adipogenic differentiation, MSCs were cultivated in basic medium containing adipogenic supplements (1 μM dexamethasone, 500 μM 3-isobutyl-1-methylxanthine, 1 μg/mL insulin, 100 μM indomethacin). Oil Red O staining was performed by culturing cells for 18 days in adipogenic medium. Then washed with PBS, fixed with 1% paraformaldehyde for 60 min, incubated twice in 100% propylene glycol (W294004, Sigma-Aldrich) for 3 min, stained with 1.5 mL heated Oil Red O (O0625, Sigma-Aldrich) for 10 min at 60 °C, incubated with 1.5 mL 85% propylene glycol for 5 min and washed twice with distilled water before analyzing the samples.

For the evaluation of mineralization, MSCs were cultured with basic medium with osteogenic supplements (100 nM dexamethasone, 10 mM β-glycerophosphate, 50 μM ascorbic acid-2-phosphate) for up to 21 days, then washed with PBS and fixed with 4% paraformaldehyde for 30 min. After two washing steps with distilled water, the cells were stained with 2% Alizarin Red S solution (A5533, Sigma-Aldrich) for 3 min, then washed twice and finally analyzed using an upright light video microscope (Olympus).

To promote chondrogenic differentiation, 2.5 × 10^5^ cells were gently centrifuged (150 × g, 5 min) in 15 ml polypropylene tubes to form a pellet. Without disturbing the pellet, the cells were cultured for four weeks in complete chondrogenic differentiation medium including 10 ng/ml TGFβ3 by feeding twice a week. After the culture period, cryo-sections were analyzed by toluidine-blue staining. The sections were fixed with ice-cold acetone and stained with 1% toluidine-blue solution (89640, Sigma-Aldrich).

### Immunofluorescence staining

For immunofluorescence assays, the cancer cells were fixed with 4% paraformaldehyde for 10 min and permeabilized with 0.3% Trion X-100 for 30 min, then blocked with goat serum (5%, Sigma-Aldrich) for 30 min, and incubated with rabbit antibodies against E-cadherin (Santa Cruz Biotechnology), Vimentin (Abcam), and rabbit antibody against N-cadherin (BD Transduction Laboratories), at appropriate dilutions overnight at 4 °C. Following, the cells were washed with PBS, and reacted with a secondary antibody labelled with Alexa Flour 488 or Alexa Flour 594 (Invitrogen, ThermoFisher). Nuclei were counterstained with 6-diamidino-2-phenylindole (DAPI, 32670, Sigma-Aldrich). Immunofluorescence was examined by fluorescence microscopy (LSM700, Zeiss, Oberkochen, Germany).

### Cell migration and invasion assays

To perform the migration and invasion assays, 24-well transwell chambers (PIEP12R48, Millipore) were used. For the invasion assay, the inserts were pre- coated with 20 μg of Matrigel (354234, BD Biocoat). The cancer cells (2 × 10^4^/chamber) were suspended in serum-free DMEM, added to the upper chamber, and in serum-free DMEM with or without conditioned medium added to the lower chamber as a chemoattractant. Then the cells were incubated for 12 h (migration) or 24 h (invasion) at 37 °C. Non-migrating or non-invading cells were removed from the top chamber using a cotton swab. The cells remained in the bottom chamber were fixed with 4% paraformaldehyde for 10 min and stained with 1% crystal violet (4466, Xiya Reagent, China) in 2% ethanol for 15 min. The cells that migrated or invaded through the membrane were visually quantified in 5–8 random fields from each membrane under a light microscope. All the experiments were performed in triplicate.

### Side population analysis and spheroids culture assay

Tumor cells were detached from a subconfluent culture by trypsinization, suspended at 1 × 10^6^ cells/mL in pre-warmed DMEM containing 2% FBS, then incubated with 3–5 μg/mL Hoechst 33342 (B2261, Sigma-Aldrich), either alone or in combination with 100 μM verapamil (V4629, Sigma-Aldrich), an ABC transporter inhibitor, in dark for 90 min at 37 °C with intermittent mixing. At the end of the staining, the cells were spun down and resuspended in cold PBS containing 5% BSA. FCM analysis and cell sorting were carried out directly on EPICS ALTRA Flow Cytosorter (Beckman Coulter, Brea, CA, USA). Hoechst 33342 was excited with a 100 mW UV laser and the signal was detected with a 450 BP filter for blue fluorescence, and with a 675 BP filter for red fluorescence. A 610 DMSP was used to separate the emission wavelengths. A polygonal live gate in a FS-HO blue plot was created to exclude debris and dead cells. Side population (SP) cells and non-SP cells were sorted for the following assays.

For spheroids culture assays, the TE-1 cells were cultured in a modified tumor sphere medium: RPMI-1640/DMEM (F12) 1:1 medium consisting of a chemically defined serum-free medium with human recombinant epidermal growth factor (EGF) (20 ng/ml, 100–47, Peprotech, Roch Hill, NJ, USA), basic fibroblast growth factor (bFGF) (20 ng/ml, 100–18B, Peprotech) and 0.5% BSA. Tumor cells were plated at a density of 2 × 10^4^ cells/well in ultralow attachment 6-well plates. Spheroids were defined as 3-dimensional cell colonies with blurred cell margins. The ability of spheroids formation was calculated by counting the spheroids number. All of the assays were performed in triplicate.

### Cell proliferation and apoptosis assays

Tumor cells were seeded in 96-well plates at a density of 5 × 10^3^ per well and allowed to recover for 12 h. The proliferation of tumor cells was evaluated using the Cell Counting Kit-8 (CK04–11, Dojindo, Japan) every 12 h. The cells were incubated with 10 μl regent for 1 h at 37 °C. Ratio of absorbance in each sample was determined by the luminescence reader (Infinite-F500, TECAN, Austria), according to the manufacturer’s instructions.

Apoptosis was detected by measuring Annexin V levels and the LIVE/DEAD Viability/Cytotoxicity Kit (L3224, Invitrogen) after treatment with chemicals. For the Annexin V assay, sub-confluent cells were cultured in 6-well plates and harvested by trypsinization without EDTA. After two washes with cold PBS, the cell pellets were resuspended in binding buffer. Annexin V (556420, BD pharmingen) and propidiumiodide (PI) staining were performed according to the manufacturer’s instructions and signals were measured by FACS.

For LIVE/DEAD Viability/Cytotoxicity Kit assay, cancer cells were cultured in 6-well plates at a density of 8 × 10^4^ per well. After 48 h of treatment, the plates were removed from the incubator and processed with a kit. After 15 min incubation at room temperature, the luminescence of each sample was examined by fluorescence microscopy. All the procedures were performed according to the kit’s protocols and the experiments were performed in triplicate.

### Animals and tumor xenograft model *in vivo*

Four-week-old male BALB/c nude mice were injected with 1 × 10^6^ tumor cells (control group) either alone or in combination with the same amount of MSCs (total 2 × 10^6^ mixed cells in the experiment group) into the left/right proximal tibia, and randomly divided into two groups (9 mice per group). The tumor volume was measured every single day until the animals were sacrificed. The tumor volume was calculated using the following equation: volume = W^2^ × L × 0.5, where W and L represent the average width and length of the tumor, respectively. Nine mice (TE-1 group) were sacrificed on day 16, and *in situ* tumor samples were collected for histological analysis. Another 9 mice (Eca109 group) were maintained until day 19 when *in situ* tumors were too large to carry on. Tumor samples from each nude mouse were fixed in 4% paraformaldehyde after sacrifice. Then the tissues were embedded in paraffin and cut into 4 μm sections for immunohistochemistry analysis. All of the experimental protocols were approved by the Animal Ethics Committee of the Sun Yat-sen University.

### Clinical specimens and immunohistochemistry staining

30 tumor samples were obtained from 30 cases of ESCC patients subjected to esophagectomy in the 1^st^ Affiliated Hospital of Sun Yat-sen University between January 2011 and August 2014, who were diagnosed with ESCC by more than two pathologists. Patient clinical parameters are summarized in Supplementary Table S3. Informed consent was obtained from each patient before participation in this study. The follow-up schedule of patients was every 3 months during the first postoperative year and at least 6 months afterward for recurrence inquiry until death or until the end of the investigation. All 30 patients were younger than 80-year old, and were first diagnosed cases without having undergone chemotherapy, radiotherapy or other treatments. This study has been approved by 1^st^ Affiliated Hospital of Sun Yat-sen University Ethics Committee, and the patient information and specimens collected have been handled anonymously according to the ethical and legal standards.

All the slides (both patients and mouse samples) were incubated at 60 °C for 1 h, deparaffinized in xylene, and rehydrated through a graded ethanol series. After antigen retrieval (boiling in the microwave for 10 min in 10 mM sodium citrate, pH 6.0), intrinsic peroxidase activity was blocked by incubation with 3% hydrogen peroxide for 15 min. Nonspecific antibody binding sites were blocked using 5% BSA. The slides were covered with appropriately diluted primary antibodies and incubated at 4 °C overnight. After three washes in PBST for 5 min each, secondary antibodies were applied for 30 min at 37 °C and staining was developed using the DAB Detection kit (kit-5010, Maxvision, China) according to the manufacturer’s instructions.

### Statistical analysis

The data are represented as the means ± standard deviations (SDs). Comparisons between two groups were performed using the Student’s *t* test, and one-way ANOVA was used for multiple comparisons. Independent *t* test was performed for comparison of data of independent samples. For the overall survival analysis, Kaplan–Meier curves were analyzed using a log rank test. A *p* value < 0.05 was considered to be statistically significant.

### Data availability

The datasets generated during and/or analysed during the current study are available from the corresponding author on reasonable request.

## Electronic supplementary material

Supplementary Information
