# Supplementary material for: Mesenchymal Stromal Cells-Derived β2-Microglobulin Promotes Epithelial–Mesenchymal Transition of Esophageal Squamous Cell Carcinoma Cells
Source: Sci Rep. 2018 Apr 3;8:5422. doi: 10.1038/s41598-018-23651-5 (PMC5883027; doi:10.1038/s41598-018-23651-5)
Supplement: Supplementary file 1 — Supplementary Information [file 41598_2018_23651_MOESM1_ESM.pdf]

Supplementary Information

Mesenchymal Stromal Cells-Derived β2-Microglobulin Promotes Epithelial–Mesenchymal Transition of Esophageal Squamous Cell Carcinoma Cells

Junjie Wang [1,#] Weilin Yang [2,3,4,#], Tao Wang [1,#], Xiaoyong Chen [1], Jiancheng Wang [1], Xiaoran Zhang [1], Chuang Cai [1], Beilong Zhong [5], Jiabin Wu [2], Zhenguang Chen [2,3,4,*], Weijun Huang[1,*] and Andy Peng Xiang [1,6,7,*]

Supplementary Figure S1: Construction of MSCs cell line with B2M knock down

Immunofluorescence study revealed the GFP fluorescence expression in MSC^{NTC} and MSC^{shB2M} cells which indirectly reflected the interference efficient of lentivirus system. Results were confirmed by qPCR and western blots analysis after passaging cells. Scales bars, 100µm.

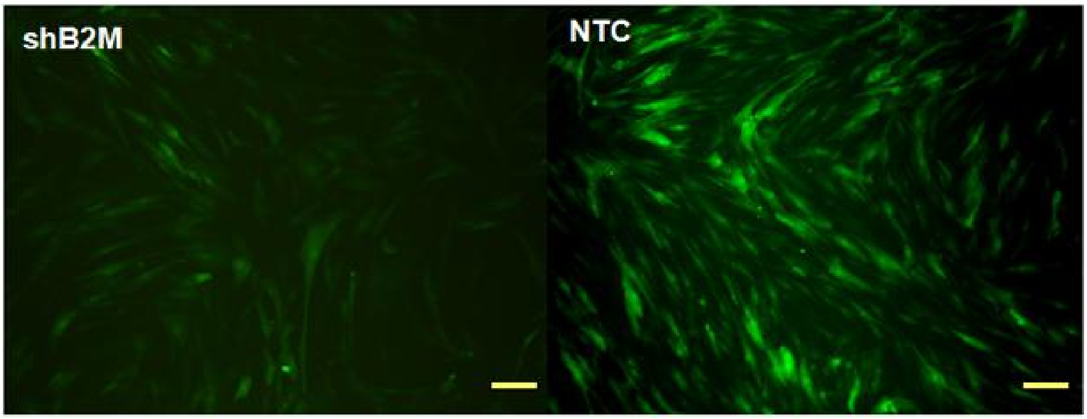

Supplementary Figure S2: Protein level of secreted B2M tested by ELISA kit

Secreted B2M level in supernatant of TE-1/Eca109/MSC cells were tested with a commercial ELISA kit (SEA260Hu, Cloud-Clone Corp). We found that protein level of secreted B2M of MSC was almost five to ten times higher than that of ESCC cell lines (TE-1: 3.75±0.34ng/ml, Eca109: 7.92±1.04ng/ml, MSC: 30.41±2.93ng/ml, data were averaged as mean ± S.D. per 10^7 cells, n = 3)

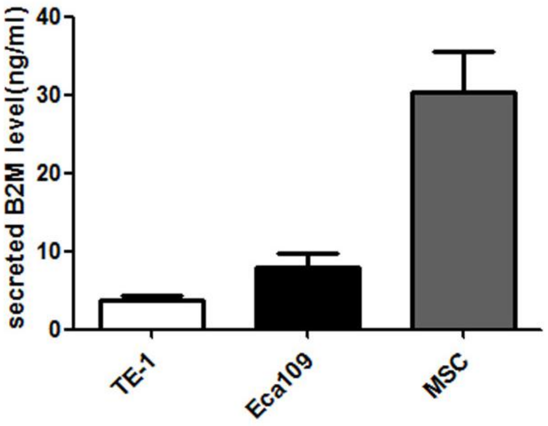

Supplementary Figure S3: Representative negative surface markers of MSC^{NTC} and MSC^{shB2M}

The results of the flow cytometry analysis revealed that MSC^{NTC} and the MSC^{shB2M} cells did not express surface markers CD34 and CD45, which was similar to wild-type MSCs.

CD34 (550761, BD Pharmingen), CD45 (560975, BD Pharmingen)

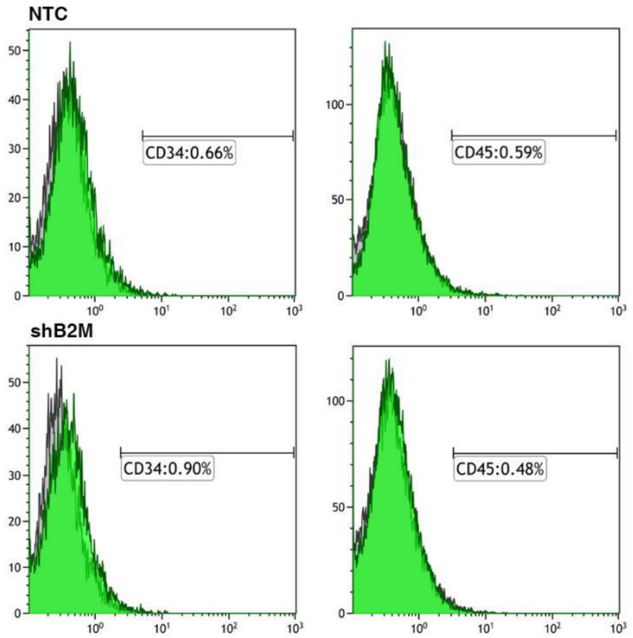

Supplementary Figure S4: Representative image of tumor spheroid

Representative image of spheroid formed by TE-1 cells in a modified serum-free medium. Spheroids which have more than 50μm in diameter are included in the statistics. Scales bars, 200μm.

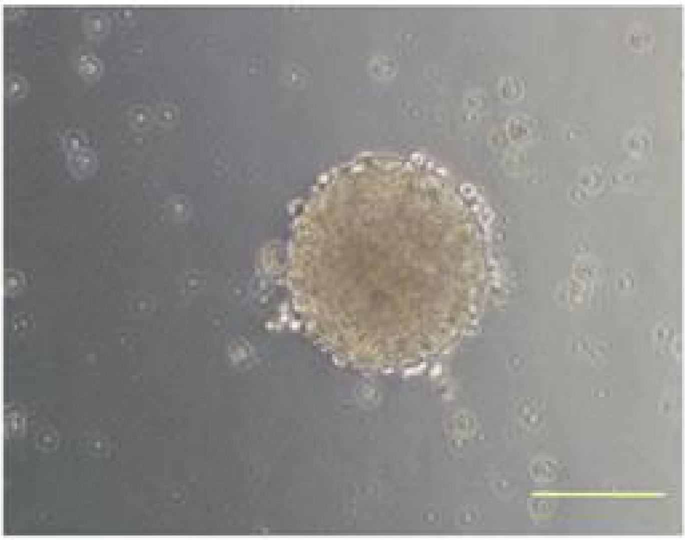

Supplementary Figure S5: Original western blot scanning document of Figure 1

Marked sections with red lines were presented in the manuscript.

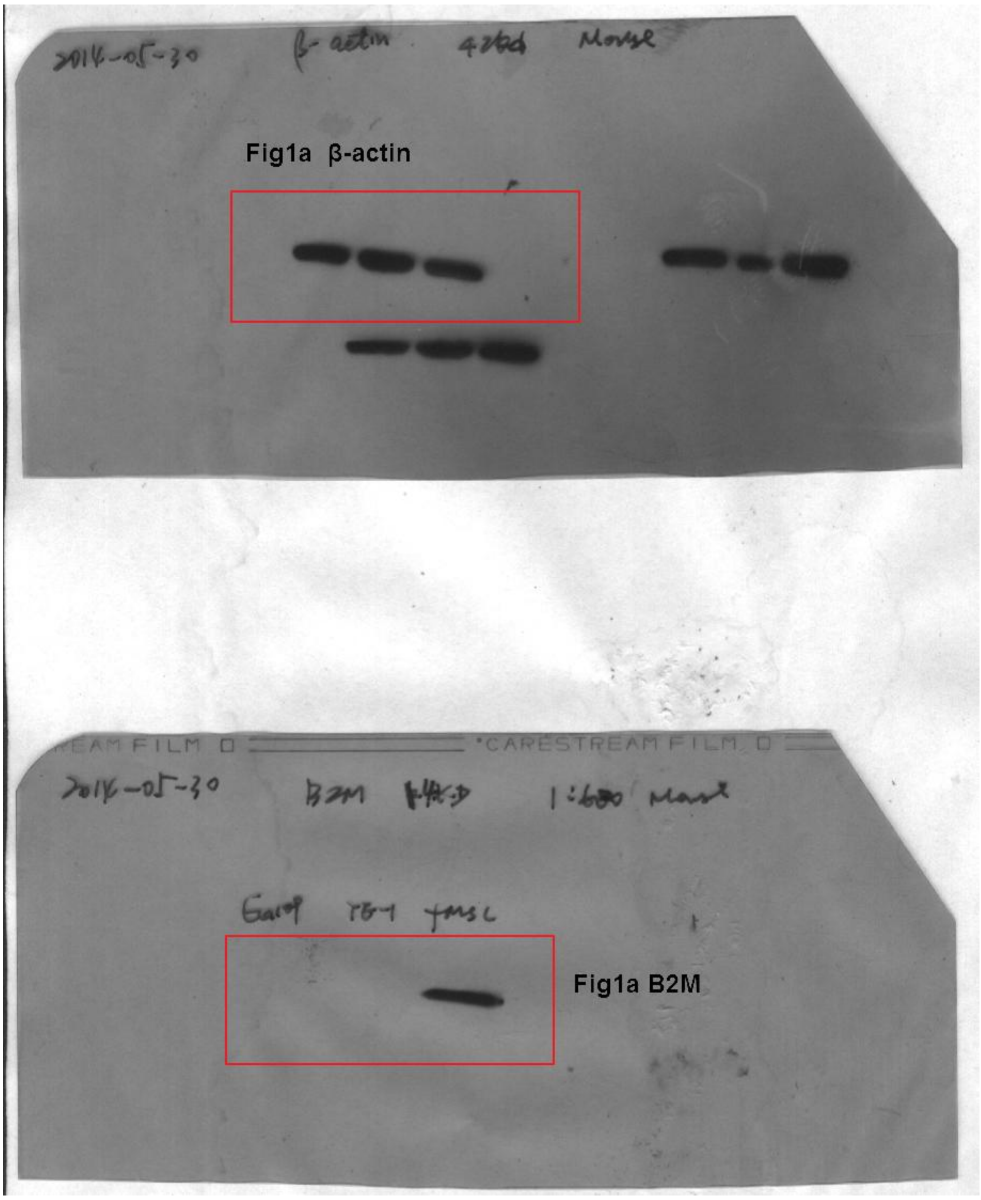

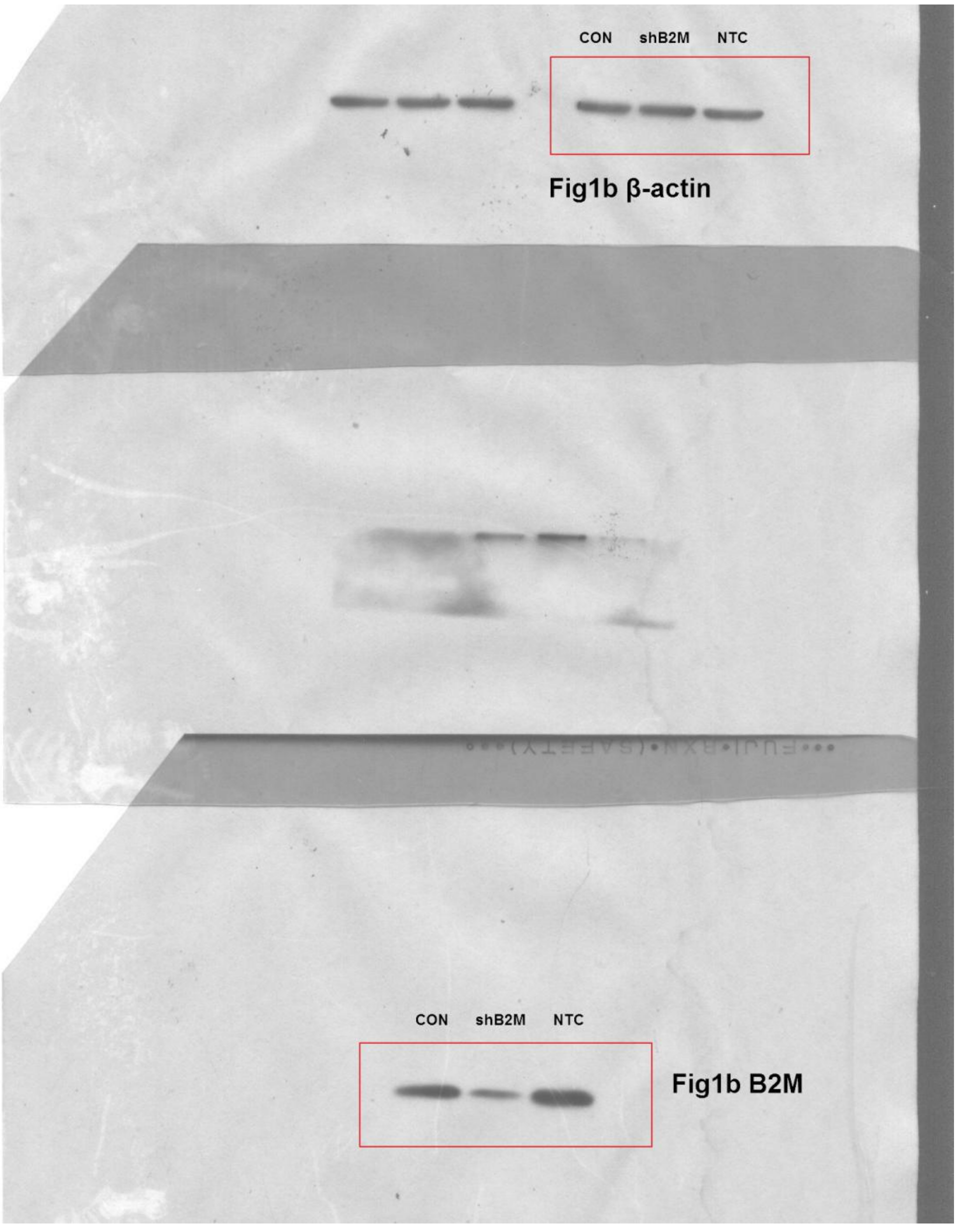

Supplementary Figure S6: Original western blot scanning document of Figure 2

Marked sections with red lines were presented in the manuscript.

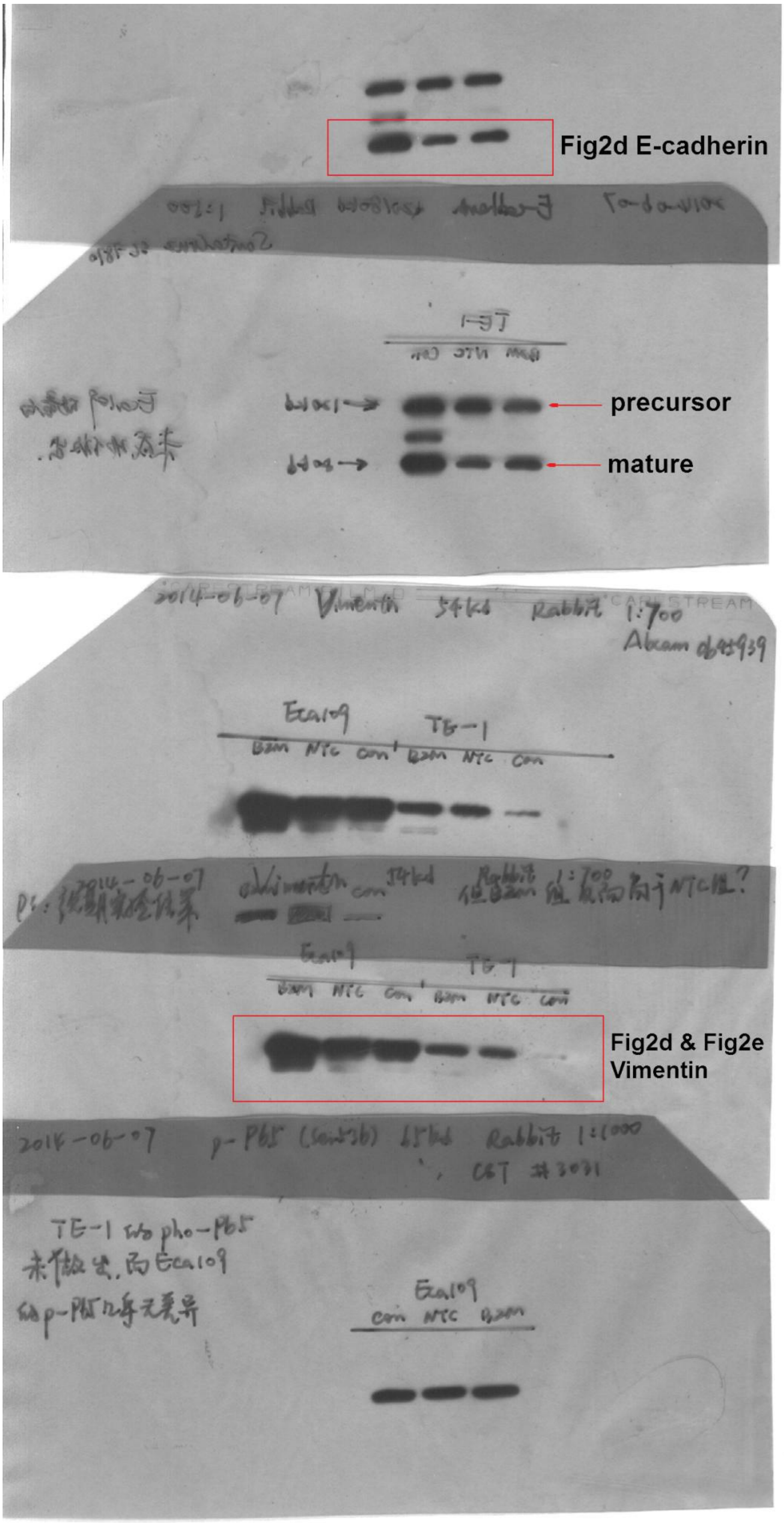
Fig2d E-cadherin
precursor
mature
2014-06-07 Vimentin 54kd Rabbit 1:700
Abcam ab45939
Eca109
TE-1
Fig2d & Fig2e
Vimentin
2014-06-07 p-P65 Rabbit 1:1000
CST #3031

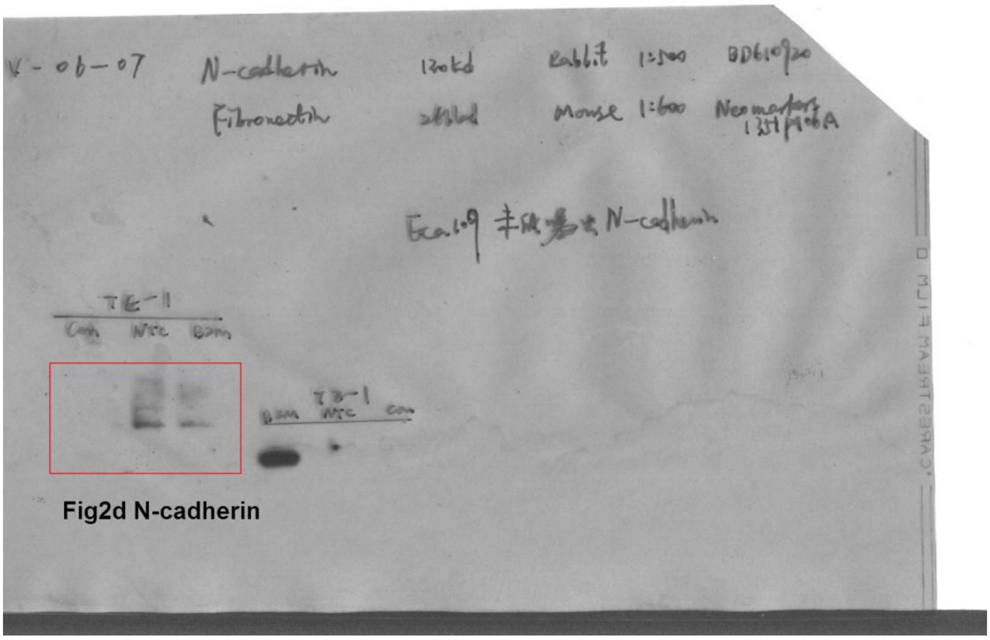

Fig2d N-cadherin

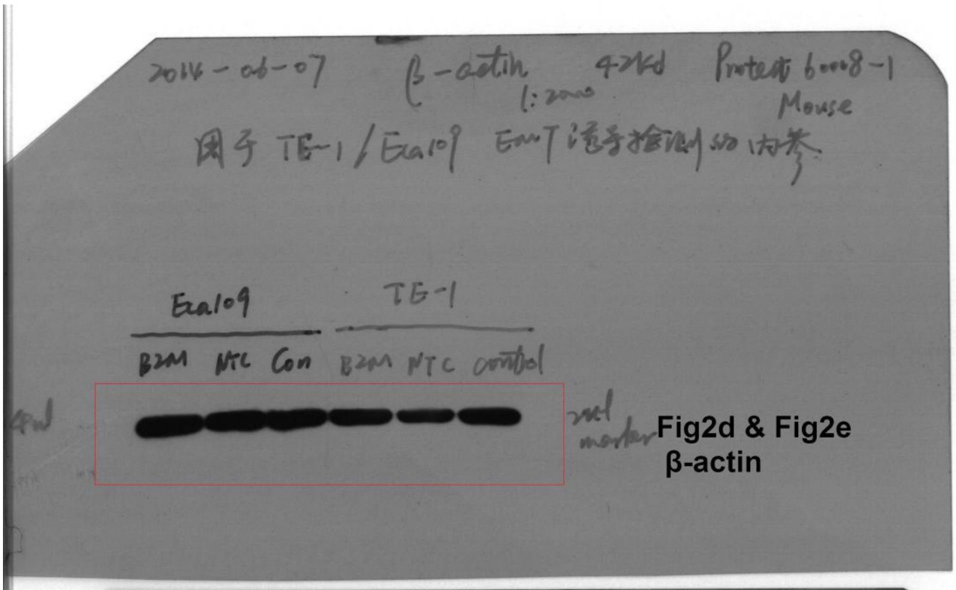

Fig2d & Fig2e β-actin

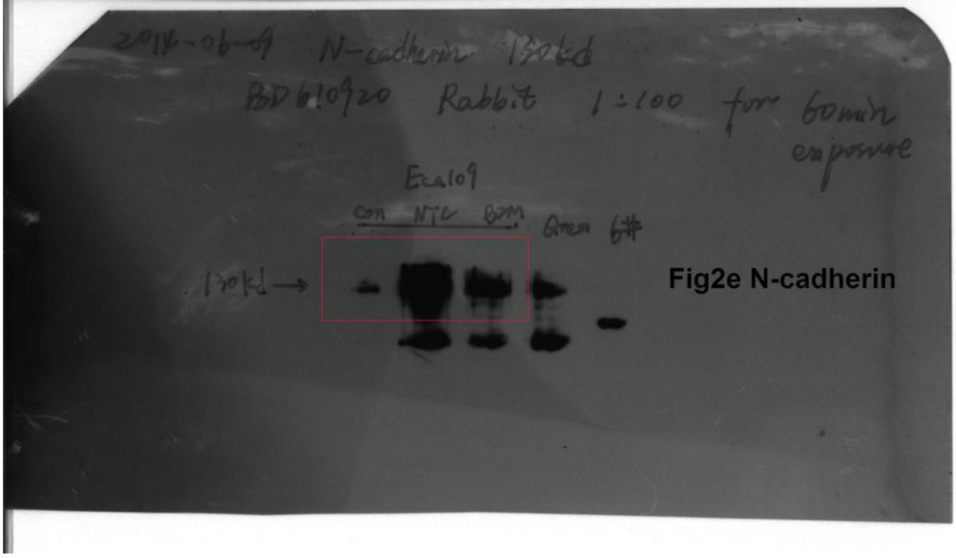

Fig2e N-cadherin

Supplementary Figure S7: JAK2 inhibitor sabotaged the EMT induction of MSC^{NTC}-CM

Western blots analysis in Eca109 cells indicated that IL6/JAK2/STAT3 pathway involved in the EMT process triggered by MSC^{NTC}-CM which could be inhibited by AG490 (T3434, Sigma-Aldrich). Blocking B2M in MSCs partially inhibited the expression of snail but barely affected phosphorylated STAT3.

Original western blot scanning document were attached.

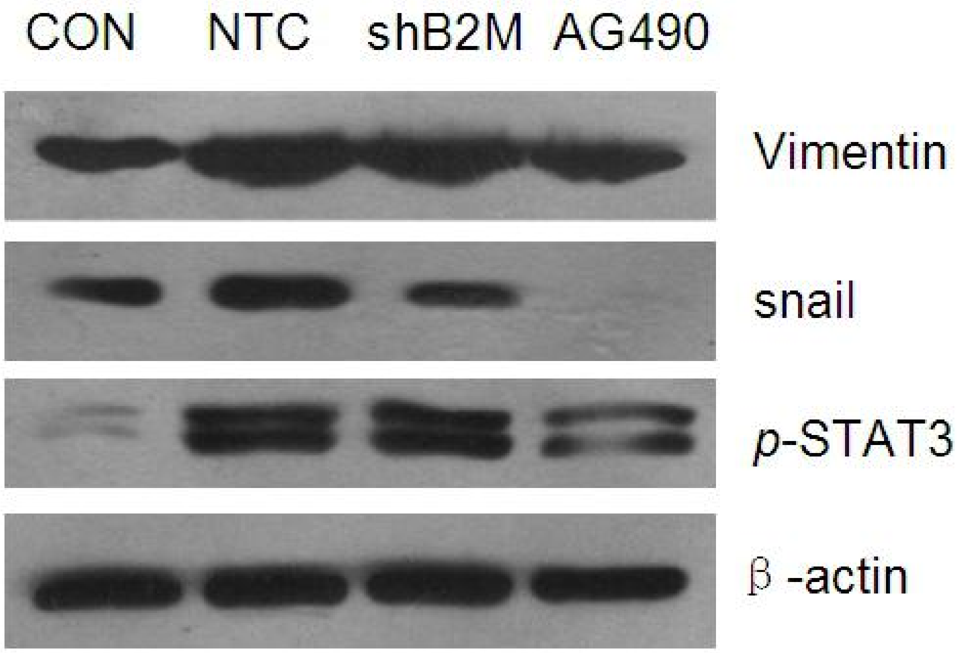

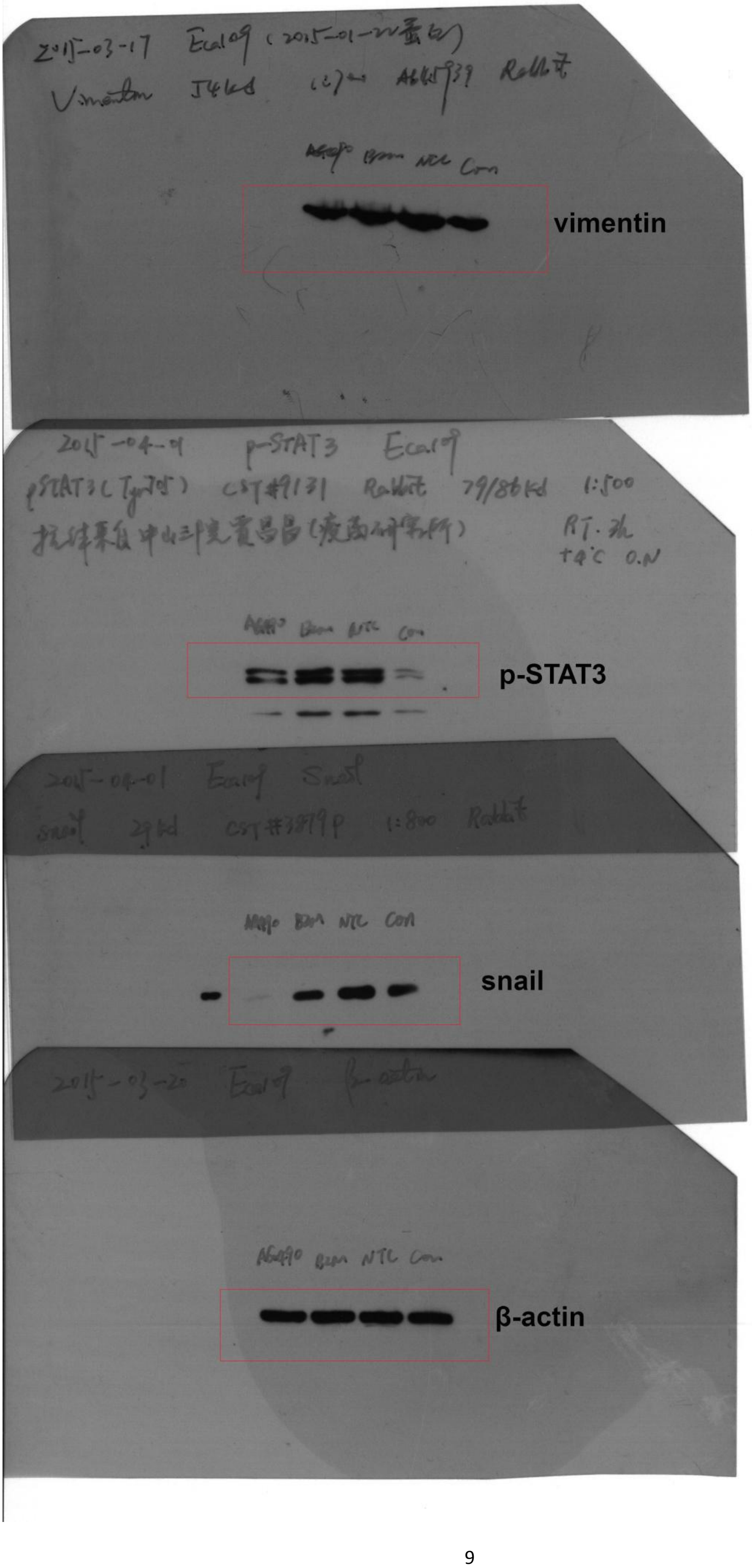
vimentin
p-STAT3
snail
β-actin

Supplementary Table S1. Sequences of oligos used for cloning shRNAs

shRNA ID	Forward /Reverse	Oligo Sequence
B2M-1	Forward Oligo	TGCCGTGTGAACCATGTGACTTTCTTCCTGTCAAAAGTCACATGGTTCACACGGTTTTTC
	Reverse Oligo	TCGAGAAAAAACCGTGTGAACCATGTGACTTTTGACAGGAAGAAAGTCACATGGTTCACACGGCA
B2M-2	Forward Oligo	TGAGGTTTGAAGATGCCGCATTTCTTCCTGTCAAAATGCGGCATCTTCAAACCTTTTTTC
	Reverse Oligo	TCGAGAAAAAAGGTTTGAAGATGCCGCATTTTGACAGGAAGAAATGCGGCATCTTCAAACCTCA

Supplementary Table S2. List of the qRT-PCR primers used in this study

Target Gene	Forward primer	Reverse primer
GAPDH	5'-GAAGGTGAAGGTCGGAGTC-3'	5'-GAAGATGGTGATGGGATTTC-3'
E-cadherin	5'-CCCACCACGTACAAGGGTC-3'	5'-CTGGGGTATTGGGGGCATC -3'
N-cadherin	5'-GCGCTGGCACCGTTTTTAC-3'	5'-CCTGAGCACGAAGAGTGTAGA-3'
Vimentin	5'-ACGTCAGCAATATGAAAGTGTG-3'	5'-ACCTGTCTCCGGTACTCA-3'
B2M	5'-TGTGCTCGCGCTACTCT-3'	5'-GTCAACTTCAATGTCGGATG-3'

Supplementary Table S3. The detail information of 30 cases esophageal cancer patients after resection.

Patient No.	Gender F/M	Age	Differentiation grade	Tumor Stage	B2M IHC result	PFS (month)
824806	F	60	Moderate	IIIB	(-)	8
828887	F	49	Moderate	IIA	(-)	14
848967	F	63	Moderate	IIIA	(-)	9
837978	F	60	Moderate	IIB	(-)	8
857081	F	53	Moderate	IIA	(-)	9
812597	F	69	Moderate	IIIA	(-)	4
824444	F	46	Moderate	IIIB	(-)	6
861532	F	51	Moderate	IIA	(-)	7
827923	F	58	High	IV	(-)	4
834531	F	57	Moderate	IIA	(-)	12
828914	F	63	High	IIIA	(-)	4
831680	F	55	Moderate	IIIA	(-)	5
838666	F	48	Moderate	IIA	(-)	10
837682	F	59	High	IIA	(-)	12
829769	F	72	Moderate	IIA	(-)	12
833668	F	59	Moderate	IIA	(-)	6

819458	F	62	High	IIA	(-)	10
828752	F	41	Moderate	IIA	(-)	10
816156	M	75	High	IIA	(+)	8
852226	F	59	Moderate	IIA	(+)	4
858149	F	60	Moderate	IIA	(+)	6
811757	F	69	Moderate	IIA	(+)	6
818960	M	57	High	IIA	(+)	2
817356	F	64	High	IIA	(+)	12
816106	M	79	Moderate	IV	(+)	6
846722	M	73	Moderate	IIA	(+)	4
827438	M	66	Moderate	IIIA	(+)	6
818887	M	62	Moderate	IIA	(+)	10
845577	F	64	Moderate	IIB	(+)	3
860896	M	54	Moderate	IIIA	(+)	2
